# The Advanced Anaerobic Expanded Granular Sludge Bed (AnaEG) Possessed Temporally and Spatially Stable Treatment Performance and Microbial Community in Treating Starch Processing Wastewater

**DOI:** 10.3389/fmicb.2018.00589

**Published:** 2018-03-28

**Authors:** Xianchao Qin, Xiaogang Wu, Lingfang Li, Chunjie Li, Zhenjia Zhang, Xiaojun Zhang

**Affiliations:** ^1^School of Environmental Science and Engineering, Shanghai Jiao Tong University, Shanghai, China; ^2^State Key Laboratory of Microbial Metabolism, School of Life Sciences and Biotechnology, Shanghai Jiao Tong University, Shanghai, China

**Keywords:** starch wastewater, AnaEG, anaerobic biodegradation, performance estimation, temporal distribution, spatial distribution, microbial community structure

## Abstract

This study implements temporal and spatial appraisals on the operational performance and corresponding microbial community structure of a full-scale advanced anaerobic expanded granular sludge bed (AnaEG) which was used to treat low organic loading starch processing wastewater. Results showed stable treatment efficiency could be maintained with long-term erratic influent quality, and a major reaction zone located at the bottom of the AnaEG, where the main pollutant removal rate was greater than 90%. Remarkably, high-throughput sequencing of 16S rRNA gene amplicons displayed that the predominant members constructed the major part of the overall microbial community and showed highly temporal stability. They were affiliated to Chloroflexi (16.4%), Proteobacteria (14.01%), Firmicutes (8.76%), Bacteroidetes (7.85%), Cloacimonetes (3.21%), Ignavibacteriae (1.80%), Synergistetes (1.11%), Thermotogae (0.98%), and Euryarchaeota (3.18%). This part of microorganism implemented the long-term stable treatment efficiency of the reactor. Simultaneously, an extraordinary spatial homogeneity in the granule physic properties and microbial community structure along the vertical direction was observed within the AnaEG. In conclusion, the microbial community structure and the bioreactor’s performance showed notable spatial and temporal consistency, and the predominant populations guaranteed a long-term favorable treatment performance of the AnaEG. It provides us with a better understanding of the mechanism of this recently proposed anaerobic reactor which was used in low organic loading wastewater treatment.

## Introduction

Starch serves as the important source for humans and industrial production, thus, it is used extensively around the world (Avancini et al., 2007). Notably, more than 20 million tons of starch processing wastewater (SPW) were generated in China per year (Lu et al., 2009). Unfortunately, the direct discharge of SPW would cause serious water pollution due to its high concentration of organic and chemical reagents. Consequently, numerous methods are applied for SPW treatment, especially, anaerobic biological treatment is the enormously popular one because of its many advantages. Such as high organic loading rate (OLR), low energy consumption, and renewable energy production.

Among the different alternatives of anaerobic biotreatment, the expanded granular sludge bed (EGSB), an improved anaerobic treatment technology based on an upflow anaerobic sludge blanket (UASB), has attracted more attention in SPW treatment in recent years (Fang et al., 2011; Fettig et al., 2013). However, its treatment performance stability is still unsatisfactory (Zhao et al., 2012). Recently, an advanced anaerobic expanded granular sludge bed (AnaEG) which combines the advantages of UASB and EGSB technology was invented by Li et al. (2014). In this new type reactor, the effluent does not need to be recycled back to the influent to maintain a high upward velocity. And well-dispersed upward influent flow increases the expansion grade of AnaEG reactor by the plug flow pattern. Furthermore, almost no surplus sludge need to dispose of the reactor and its adaptability to shock-load are higher than other anaerobic reactor. Consequently, the AnaEG overcomes the shortcomings of the existing anaerobic reactors. Its high efficient decomposition of organic pollutant plus remarkably reduced energy consumption displays the big advantage of this reactor. Anaerobic digestion is characteristic for its application on high concentration organic wastewater (Li and Yu, 2016). However, some researchers found that the anaerobic bioreactor also could complete excellent treatment performance for the low OLR [less than 1 kg chemical oxygen demand (COD) m^-3^ d^-1^] industrial wastewater processing (Zhou et al., 2009). And the AnaEG had been efficiently operated for treating low organic loading wastewater in lab-scale experiment (0.806 kg COD m^-3^ d^-1^; Li et al., 2014), and also has been implemented in various industrial wastewater treatment, including SPW, ethanol wastewater, pharmaceutical and chemical wastewater. The factories’ monitoring data demonstrated that high treatment efficiency could be achieved by AnaEG (data not published). Thus, the AnaEG has potential for prevail usage in various organic wastewater treatment. It might represent a new generation of anaerobic wastewater treatment processes.

More important, a comprehensive knowledge to the microbial community structure of anaerobic reactor is crucial for the industrial application. Because it is not only paramount to understanding the potentials and limitations of the metabolic reactions (McKeown et al., 2012), but could also be used to take precautions to prevent inhibitory processes that have the tendency to significantly disturb anaerobic bioreactor (Niu et al., 2016). In the existed microbiological studies, some of them indicated that the bacterial and archaeal populations demonstrated a temporal succession when the psychrophilic anaerobic reactor was operated at increasing loading rate and VFA accumulation condition (Connaughton et al., 2006). Other researchers indicated that different microbial populations could be observed in the primary and secondary metabolism of quinoline degradation (Wang et al., 2017). However, others found that the bacterial diversity was quite chaotic but the archaea remained relatively stable over the whole operation period (Perendeci et al., 2013). Furthermore, because anaerobic digestion is a successive metabolic procession, and different microbial communities are responsible for these metabolisms. Hence, researches revealed that microbial spatial distribution existing in anaerobic reactor. For example, a stratified microbial structure was detected in the anaerobic reactor (Antwi et al., 2017) and the methanogenic, acetogenic, and hydrolytic fermentative microorganisms were predominant in the upper, middle, and bottom compartments of the reactor, respectively (Xing et al., 2014). In addition, researchers found that some groups could be enriched in the special environment. For example, in a study on EGSB for sucrose wastewater treatment, Firmicutes and a H_2_-utilizing methanogen, *Methanospirillum*, significantly increased when the temperature gradually decreased from 20 to 10°C (Tsushima et al., 2010). And others indicated that the shock load reduced the abundance of hydrogenotrophic methanogenic Methanomicrobiales, but dramatically increased the abundance of Methanobacteriales (Bialek et al., 2013).

In the case of AnaEG reactor, there is few study for the microbial community. Only a briefly description in the research of Li et al. (2014), when AnaEG was used to treat coal gasification wastewater, they found that micrococci were the main microorganisms and filamentous bacteria intertwined randomly throughout the cross-section. It is regrettable that they did not make an in-depth analysis of the specific composition and distribution of these microorganisms. To the best of our knowledge, there is no other comprehensive investigation of microbial community structure on both temporal and spatial dimension of the anaerobic bioreactor, especially on the AnaEG bioreactor. Considering the unparalleled merits of AnaEG, it is worth investigating the microbial community when it was used to low organic loading wastewater processing.

Hence, in this study, we investigated the performance of a full scale AnaEG reactor. The main goals in this study were to (i) assess the temporal and spatial degradation performance of a long-term operational full-scale AnaEG for SPW treatment and (ii) clarify the temporal and spatial microbial community structure that was responsible for the bioreactor’s treatment performance.

## Materials and Methods

### Reactor Description and Wastewater Characteristics

The bioreactor used in this study was a full-scale AnaEG reactor (Supplementary Figure S1) which was used in a modified starch wastewater treatment plant located in Hangzhou, Zhejiang Province of China. It has been operated for more than 8 years. The internal diameter of this reactor is 8 m and with the height of 15 m, the total volume is 750 m^3^ (Supplementary Figure S1A). Seven sampling ports (H1–H7) are vertically distributed on the reactor from the bottom to the middle area (Supplementary Figure S1C). The respective heights of these ports are 1, 2, 3, 4, 5, 7, and 9 m, and the upper part of the reactor has a gas–liquid–solids separator (Supplementary Figure S1B). The grade of sludge expansion in the AnaEG depends upon the influent and the biogas production, and is automatically adjusted by the loading rate. The effluent does not need to be recycled back to the influent to maintain a high upward velocity, and the wastewater flows upward in a plug flow pattern.

The main raw material for this starch wastewater treatment plant was cassava, potato, and maize starch. Depending on the product desired, one or more types of these raw materials were used to modify the starch for production. Other chemical additives, such as sodium hypochlorite, sodium trimetaphosphate, and acetic anhydride, were used in the modifying process according to the demands of the product. Wastewater was first collected in a batching pool after pretreatment and then introduced to the AnaEG. The temperature of the SPW was maintained at 30–35°C by steam heating, and the pH ranged from 5.7 to 8.5. The characteristics of the wastewater in terms of its COD, ammonium (NH_4_^+^), total nitrogen (TN), total phosphorus (TP), chloride (Cl^-^), and suspended solids (SS) are shown in **Table 1**.

**Table 1:** Physicochemical parameters of influent.

Influent parameter	Range of variation
pH	5.7–8.5
Temperature (°C)	30–35
COD (mg L^-1^)	2,953–9,483
NH_4_^+^-N (mg L^-1^)	1.1–10.2
TN (mg L^-1^)	9.04–22.8
TP (mg L^-1^)	12.4–200
Cl^-^ (mg L^-1^)	427–1,958
SS (mg L^-1^)	200–1,050

### Operation Parameters of the AnaEG and Sample Collection

The upflow rate of the AnaEG was 6–15 m^3^ h^-1^, corresponding to 0.12–0.3 m h^-1^ upflow velocity and 2–5 days of hydraulic retention time (HRT). The OLR ranged from 1 to 3.3 kg COD m^-3^ d^-1^. Wastewater sampled from the batch pool and mid-sedimentation tank served as influent and effluent, respectively. The interior wastewater of the reactor was also collected from the seven sampling ports that were located at different heights of the bioreactor (H1–H7) to appraise the reactor’s treatment performance. Anaerobic granular sludge (AGS) samples were collected from the seven sampling ports to analyze the microbial community structure. The sampling intervals varied from 2 to 6 weeks for 4 months from March through June 2016. A total of 31 sludge samples were obtained from five sampling time points (T1–T5). After sample collection, the sludge water mixed samples were first centrifuged at 10,000 ×*g* for 5 min to separate the supernatant and sludge for further analysis. All the samples were temporarily stored at 4°C, and the sludge for the microbial community analysis was stored at -80°C before the experiments were carried out. The pH, temperature, COD, TOC, and VFAs were analyzed from the wastewater samples to evaluate the treatment performance. AGS characteristics, including MLSS, MLVSS, settling volume (SV), granule size, and scanning electron microscopy (SEM) observation, were investigated as well. High-throughput sequencing was implemented for the AGS to examine the temporal and spatial variation dynamics of the microbial community structure in the AnaEG.

### Analytical Methods

Parameters, including pH, temperature, COD, TOC, MLSS, MLVSS, and SV analysis, were examined as described previously (Li et al., 2016). Granule size distribution was measured by a laser particle size analyzer (Mastersizer 3000, United Kingdom). VFAs were determined by ultra-fast liquid chromatography (UFLC) equipped with an LC-20AD binary gradient pump, a DGU-20A degasser, a UV-VIS detector (SPD-10A), a CTO-20AC column oven and controlled by a workstation (model CBM-20A) with data acquisition system LC Solutions software. The stationary phase was a C18 column (150 mm × 4.6 mm), and the mobile phase consisted of 10 mM KH_2_PO_4_:methanol (75:25, v/v) with a flow rate of 1 mL min^-1^. A 20 μL sample solution was injected into the UFLC system, and the column oven was kept at 30°C. The wavelength was 210 nm, and the VFAs were identified by the retention time by comparing them with standards after 15 min of chromatography. All the equipment was manufactured by the Shimadzu Corporation (Kyoto, Japan), and all the reagents were HPLC grade (Aladdin, United States). Analysis of variance (ANOVA) of Kruskal–Wallis test was used to determine the physicochemical data variance, and the differences were considered significant when *P* < 0.05.

### Morphological Analysis of Anaerobic Granular Sludge

The AGS for SEM observation were first soaked in 2.5% glutaraldehyde overnight at room temperature in the dark, and then the solution was discarded. Next, the AGS was gradient dehydrated in a series of 30, 50, 70, 90, and 100% (v/v) absolute ethanol solutions for 15 min each time. One hundred percent v/v dehydration was repeated three times. And then, the AGS samples were dried by a liquid carbon dioxide critical evaporator (Quorum, United Kingdom), and metal spraying was conducted using an ion sputtering apparatus (Leica, Germany). Finally, the samples were observed with SEM (Hitachi, Japan).

### Microbial Community Analysis

#### Preparation of the 16S rRNA Gene Sequencing Library

The microbial community structure of AGS was identified by high-throughput sequencing to clarify the temporal and spatial variation of the microbial community. Genomic DNA extraction of each AGS sample was conducted as described previously (Griffiths et al., 2000; Paulin et al., 2013). The V3–V4 regions in the 16S rRNA gene sequencing library were established by two-step PCR amplification according to Illumina’s instructions. Primer sets of 341F (5′-CCTACACGACGCTCTTCCGATCTNCCTACGGGNGGCWG CAG-3′) and 805R (5′-GACTGGAGTTCCTTGGCACCCGAG AATTCCAGACTACHVGGGTATCTAATCC-3′) with Illumina MiSeq adaptor sequences were used in the first step of PCR amplification. Dual index sequences were then attached to the first-step PCR amplification products by an additional round of amplification to separate the individual samples. The PCR cycle parameters were set as follows: initial denaturation at 95°C for 3 min, 21 cycles (the second round PCR was eight cycles) followed by denaturation at 94°C for 30 s, annealing at 55°C for 30 s, extension at 72°C for 30 s, and then a final extension step at 72°C for 5 min. The purified PCR products were mixed at equal ratios for sequencing using the Illumina MiSeq System (Illumina Inc., United States).

#### Bioinformatics Analysis of the Sequencing Data

The preliminary process of the raw sequencing data was conducted as per the previous document (Zhang et al., 2016). Sequence assembly was first implemented, and the unique sequences obtained by dereplication were sorted by decreasing abundance, and then singletons were abandoned. UPARSE’s default (Edgar, 2013) was used to select the representative operational taxonomy units (OTUs), and UCHIME (Edgar et al., 2011) was selected to further perform reference-based chimera detection against the RDP classifier training database (v9) (Cole et al., 2014). Finally, the OTU table was completed by mapping quality-filtered reads to the representative OTUs with Usearch (Edgar, 2010), resulting in a global alignment algorithm at a 97% cutoff. Next, the sequences of all the samples were downsized to 20,000 (1,000 permutations) to avoid spurious variation caused by different sequencing depths. Further analysis was performed using the QIIME platform (version 1.8; Caporaso et al., 2010), and then the alpha and beta diversity were determined. In addition, representative sequences for each OTU were used to establish the phylogenetic tree by FastTree and submitted to the online RDP classifier^1^ (RDP database version 2.10) to determine the phylogeny, with a bootstrap cutoff of 80%. Subsequently, the sequence abundances in the OTU table were used for the taxonomical statistics of each sample.

#### Statistical Analysis of Microbial Community

The sequencing quality of the alpha diversity analysis was evaluated by observing the OTUs and the Shannon diversity. Venn diagrams were used to depict the overall OTU distribution in five sampling time points and seven sampling heights of AnaEG reactor, simultaneously displaying the phylum that had a relative abundance greater than 1%. All the OTUs were divided into three types according to their relative abundance to explore the corresponding roles of each part of the bacterial consortium in the bioreactor’s stable operation and overall microbial community structure variation. OTUs with a relative abundance higher than 1% were classified as abundant OTUs. Those with a relative abundance higher than 0.1% but less than 1% were defined as common OTUs, and the remainder were classified as rare OTUs. ANOVA analysis was used to analyze the variation in these three types of OTUs. Furthermore, the abundant OTUs also were regarded as predominant OTUs, and its heatmap was plotted to investigate its temporal and spatial abundance changes.

#### Sequence Data Accession Numbers

The 16S rRNA gene sequences in this study were submitted to the GenBank Sequence Read Archive database under the accession number SRP111625.

## Results

### Performance of AnaEG for SPW Treatment

**FIGURE 1:**
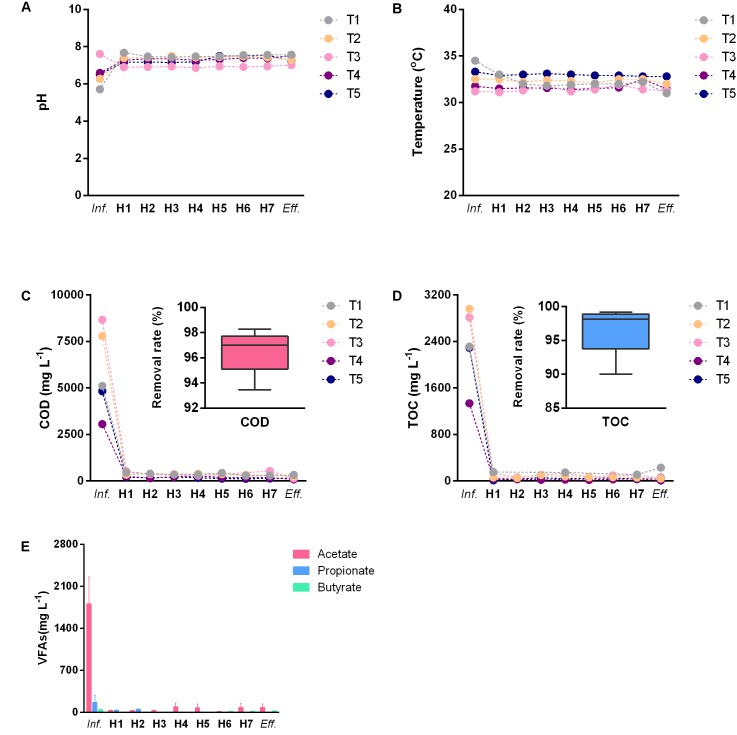
Wastewater treatment performance of AnaEG. **(A)** pH of wastewater. **(B)** Temperature of wastewater. **(C)** COD concentration and removal rate. **(D)** TOC concentration and removal rate. **(E)** VFAs of wastewater. Abbreviations of *Inf.* and *Eff.* represented influent and effluent, T1–T5 represented the five sampling time points, H1–H7 represented the seven sampling heights of the reactor.

Treatment performance estimation results of the AnaEG were shown in **Figure 1**. In term of different sampling time points, the influent pH fluctuated between 5.7 and 7.6 due to the variation of the wastewater production (**Figure 1A**). The temperature displayed minor variations of 31–34.5°C under the steam heating control (**Figure 1B**). Furthermore, COD and TOC of the influent fluctuated drastically among the different sampling time points, varying from 3,058 to 8,667 mg L^-1^ and from 1,338 to 2,964 mg L^-1^, respectively. But in any concentration, the reactor showed the removal rate higher than 96% at all of the sampling time points (**Figures 1C,D**). In term of vertical distribution of water quality parameters, no large variations of pH and temperature (lower than 1) were observed in the interior of the reactor at the different sampling ports (**Figures 1A,B**). Intriguingly, both of the COD and TOC dropped to an extremely low level before the first sampling port of the AnaEG. The reactor’s bottom area should be a highly active function zone of pollutants degradation. Moreover, influent VFAs concentrations were also at a high level, for average total VFAs more than 2,100 mg L^-1^. In particular, acetate was the most dominant component compared to propionate and butyrate, it accounted for approximately 93% of the total VFAs attributed to acetic anhydride addition in the starch modification. Similarly, all the measured residual VFAs were lower than 100 mg L^-1^ after the degradation at the bottom area (**Figure 1E**). This was particularly true for propionate and butyrate, which were lower than 20 mg L^-1^. The total VFAs were lower than 150 mg L^-1^ in all the H1–H7 port samples and effluent. All these results reflected that the AnaEG could maintain excellent pollutants degradation efficiency during a long-term operation, even with fluctuating influent.

### Characteristics of Anaerobic Granular Sludge

**FIGURE 2:**
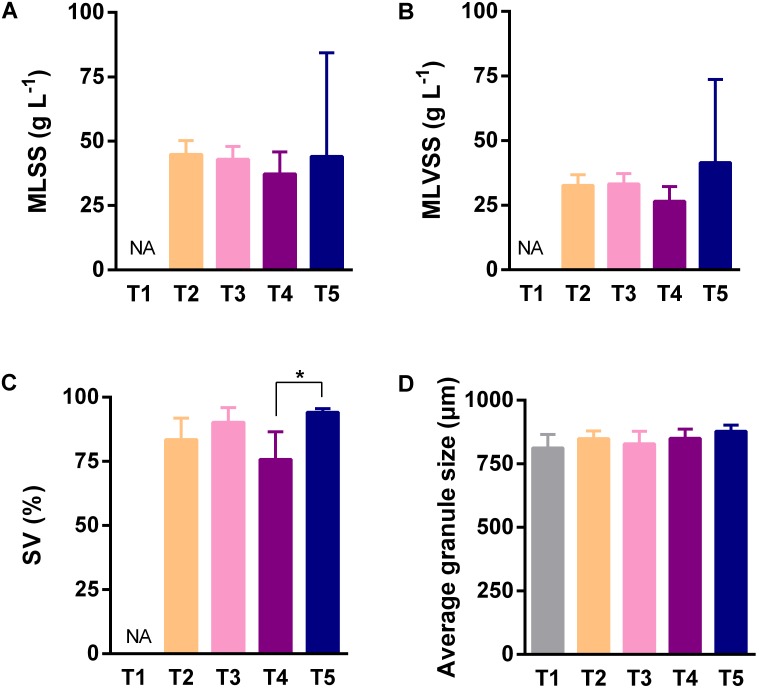
Characteristics of anaerobic granular sludge (AGS) of different sampling time points. **(A)** The mixed liquid suspended solids of sludge. **(B)** The mixed liquor volatile suspended solids of sludge (biomass). **(C)** The percentage of sludge volume. **(D)** The average diameter of granule. Error bar was SD obtained based on the data of five sampling time points, ANOVA analysis of Kruskal–Wallis test was used to analyze variation between different sampling heights, ^∗^*P* < 0.05. NA indicated no analysis.

**FIGURE 3:**
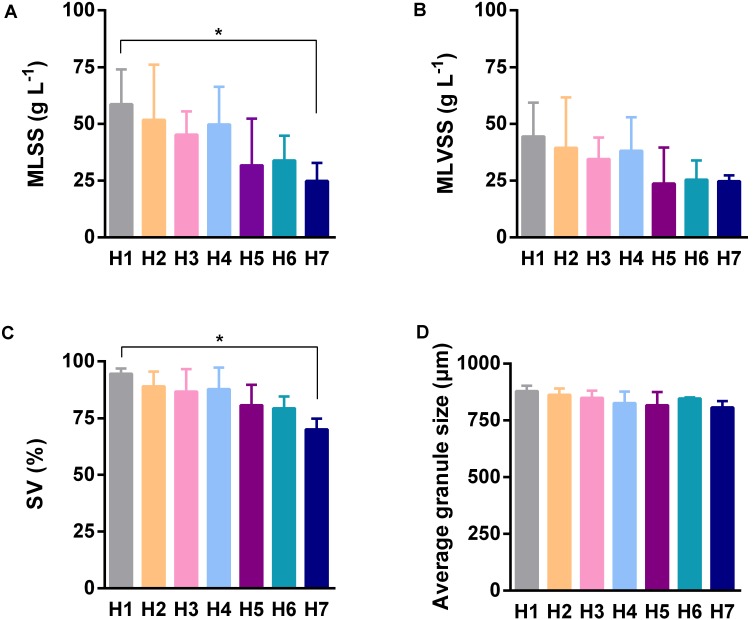
Characteristics of AGS of different sampling heights. **(A)** The mixed liquid suspended solids of sludge. **(B)** The mixed liquor volatile suspended solids of sludge (biomass). **(C)** The percentage of sludge volume. **(D)** The average diameter of granule. Error bar was SD obtained based on the five times sampling data, ANOVA of Kruskal–Wallis test was used to analyze variation between different sampling heights, ^∗^*P* < 0.05.

Because the AGS is a functional material for completing pollutant biodegradation in anaerobic bioreactors, we investigated its detail physicochemical characteristics in different sampling time points and sampling heights. In different sampling time points, the only significant difference of SV was observed between the fourth and the fifth sampling (**Figure 2C**), however, MLSS (**Figure 2A**), MLVSS (**Figure 2B**), and granule size (**Figure 2D**) did not showed statistically significant difference. In different sampling heights, MLSS (**Figure 3A**), MLVSS (**Figure 3B**), and SV (**Figure 3C**) gradually decreased from the first (H1) to the last sampling port (H7). Particularly, MLSS and SV differed significantly between the first sampling port and the seventh. In addition, the diameter of the AGS, average in approximately 900 μm, was no significant difference among the seven sampling ports (**Figure 3D**). These results indicated that both the AGS concentration and biomass were reduced vertically along the AnaEG reactor. More important, the smaller granule size and strong uniformity in this reactor may enable the implementation of a high substrate-microorganism contact. The SEM observation of the appearance indicated AGS was smoothly black spherical or elliptical granule (Supplementary Figure S2). It had a regular boundary, without obvious cavities and cracks were observed on the surface. Abundant filamentous- and cocci-shaped microorganisms constructed the surface matrix. The morphology of AGS was no obvious difference among the seven sampling ports. Moreover, cut plane SEM observations revealed that different types of microorganisms intertwined randomly throughout the cross-section. Cocci- and rod-shaped microorganisms could also be observed in the granules. Previous research suggests that they could belong to *Methanosarcina* and *Methanosaeta* (Nizami and Murphy, 2011). Although various microorganisms were observed in the different granules, the overall morphology indicated that the AGS was composed of filamentous- and bacillus-shaped microorganisms.

### Microbial Community Characteristics of the AnaEG Reactor

#### Similarity of Overall Microbial Community Structure

A total of 870,600 raw sequences were produced by the 16S rRNA gene V3–V4 region sequencing, and 846,963 high-quality sequences were obtained after pretreatment for the total 31 samples, with an average of 27,321 per sample. A total of 1,648 representative OTUs were acquired by the QIIME platform. Alpha diversity analysis showed that the Shannon diversity reached a plateau with the current sequencing level. Most of the microbial diversity had been measured (Supplementary Figure S3A), although there was no plateau of the rarefaction curve (Supplementary Figure S3B). This indicated the high quality of the sequencing data that was appropriate for subsequent analysis. The community diversity, as quantified by the Shannon index, which ranged from 6.28 to 6.63, showed temporally significant differences only between the second and fourth sampling time points (Supplementary Figure S4A). However, no significant spatial differences were observed in all the sampling heights (Supplementary Figure S4B).

**FIGURE 4:**
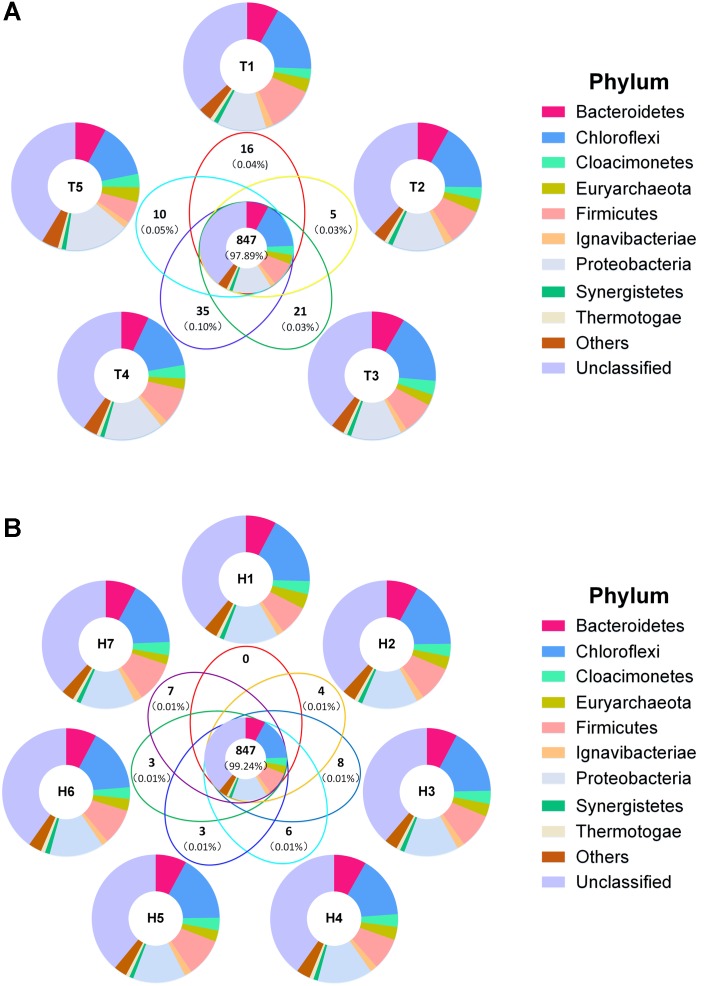
Venn diagram of total OTUs. **(A)** Venn diagram of sample by time grouping. **(B)** Venn diagram of sample by height grouping. Bold numbers represented shared and unique OTUs numbers, numbers in brackets were average relative abundance. Different colors represented phylum distribution in corresponding parts.

Venn diagram displayed the overall OTU distribution in different sampling time points and sampling heights (**Figures 4A,B**). Among the different sampling time points, total 1,243, 1,269, 1,391, 1,417, and 1,208 OTUs were detected respectively in the five time points. Notably, 847 of them were shared OTUs and occupied approximately 98% of the relative abundance of the whole sample (**Figure 4A**). However, the unique OTU numbers which existed respectively in the five time points were 16, 5, 21, 35, and 10, and all the relative abundance of them were less than 0.2%. Obviously, most of the microorganisms maintained a temporal stabilization, while few microorganisms varied with time changing. Besides, dominant phyla distribution illustrated a high similarity between their shared OTUs and each sampling time point. This attested that the AnaEG hosts a stable microbial community structure in a long-term operation. In addition, 1,252, 1,317, 1,366, 1,345, 1,282, 1,233, and 1,290 OTUs were detected respectively in the seven sampling heights, and 847 shared OTUs also were observed with relative abundance (99.24%). And 0, 4, 8, 6, 3, 3, and 7 unique OTUs were found respectively in the seven sampling heights. Moreover, the detected dominant phyla of corresponding OTUs displayed high similarity between the shared and each sampling port. Their composition and relative abundance were the same as temporal distribution (**Figure 4B**). These results implied that the microbial community structure displayed highly temporal stabilization and spatial uniformity.

#### Stabilization of Predominant Microorganisms

**FIGURE 5:**
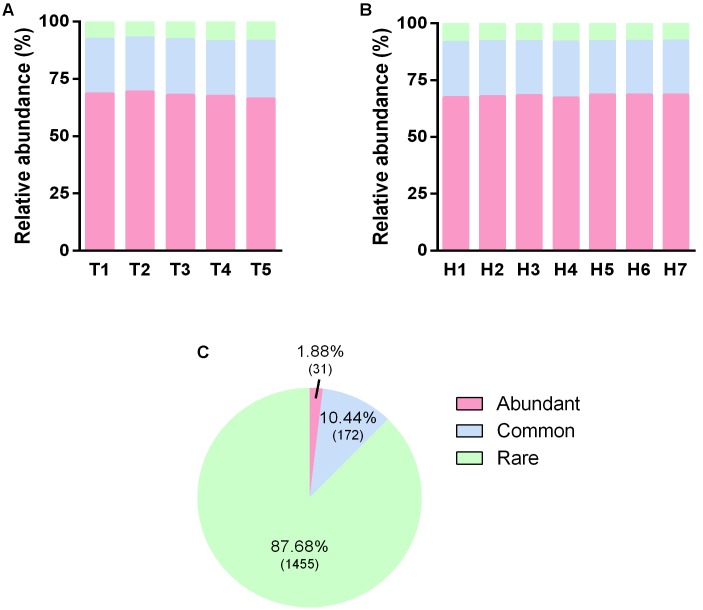
Distribution of OTUs with different occurrence frequency. **(A)** Relative abundance of OTUs with different occurrence frequency in five sampling time points. **(B)** Relative abundance of OTUs with different occurrence frequency in seven sampling heights. **(C)** The percentage of OTUs number with different occurrence frequency, the according numbers of OTUs were presented in the brackets.

In order to clarify the functional sustainer for the treatment stabilization and the efficient vertical pollutant degradation, we divided the overall OTUs into three categories, i.e., abundant OTUs, common OTUs, and rare OTUs, according to its relative abundance (see description in section “Materials and Methods”). The average relative abundance of these three type OTUs were 68, 24, and 8% (**Figures 5A,B**), but the number of them to total OTU number were 1.88, 10.44, and 87.68%, respectively (**Figure 5C**). No differences were observed for different sampling time points (**Figure 5A**) and sampling heights (**Figure 5B**). This reflects the fact that most of the microbial biomass came from some fewer type predominant microorganisms. Whereas, enormous rare microorganisms merely composed of a small part of the overall microbial community.

**FIGURE 6:**
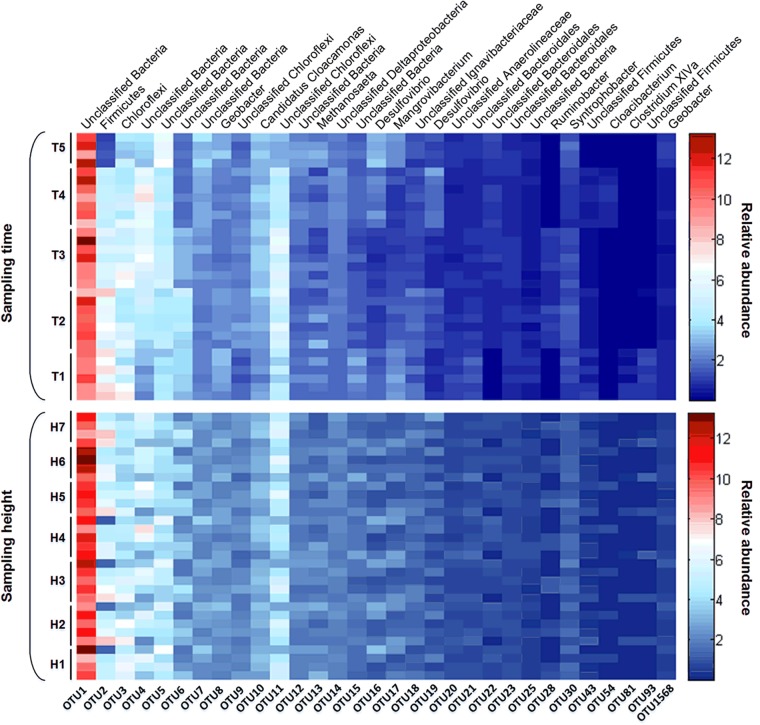
Heatmap of dominant OTUs. The relative abundance and taxonomic information of dominant OTUs in different time points and sampling heights were displayed as upper and below figures.

Furthermore, due to the important status of dominant members in the overall community of this AnaEG reactor, we further investigated the temporal and spatial varying condition of abundant OTUs. The relative abundance of abundant OTUs were showed by heatmap (**Figure 6**). Firstly, most of the abundant OTUs did not exhibit significant difference with time changing, only few of them changed in the fifth sampling time, such as OTU2, OTU4, and OTU11 reduced but OTU5 was enriched in the last sampling. Secondly, all the abundant OTUs did not demonstrate spatial varying of relative abundance, it showed a uniform distribution from the first to the last sampling ports. All the results indicated that the dominant community maintained extreme stability and uniformity in temporal and spatial dimension, respectively.

#### Taxonomic Classification of Microbial Community

**FIGURE 7:**
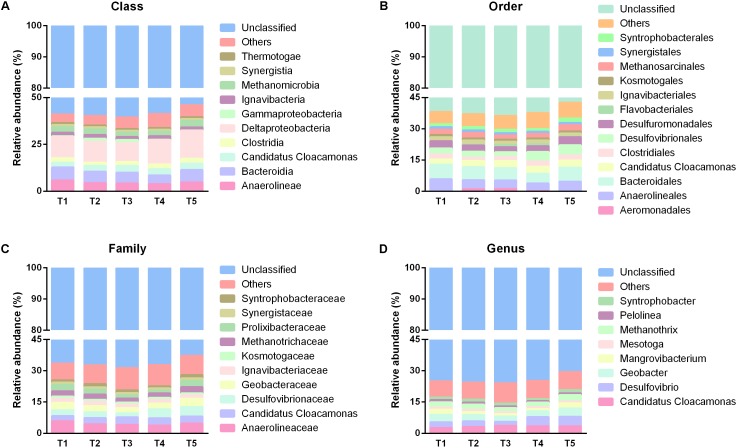
Taxonomic distribution of different time points from class to genus. **(A–D)** Were the taxonomic information of predominant populations in class, order, family and genus level, respectively.

To uncover the temporal and spatial microbial response to SPW treatment performance of the AnaEG, sequences derived from all the 31 samples were classified into different taxa, including phylum, class, order, family, and genus. The corresponding relative abundance were depicted in **Figures 5**, **6**, for phylum level, it was displayed in **Figure 2**. Notably, large proportion were unclassified taxon in this AnaEG reactor. In detail, about 38.81, 58.46, 61.75, 66.51, and 74.42% of the sequences could not be classified to phylum, class, order, family, and genus level, respectively. This phenomenon of high abundant unclassified members need to implement further study to clarify their taxonomic information and to understand their function. From phylum to genus level, the identified taxa number was 37, 64, 92, 160, and 209, respectively. The major phylogenetic groups (relative abundance higher than 1% at least in one group) of five sampling time points were illustrated in **Figures 4A**, **7**. The nine dominant phyla in all 31 samples were members of Chloroflexi (16.40%), Proteobacteria (14.01%), Firmicutes (8.76%), Bacteroidetes (7.85%), Cloacimonetes (3.21%), Ignavibacteriae (1.8%), Synergistetes (1.11%), Thermotogae (0.98%), and Euryarchaeota (3.18%), and no significantly difference was observed among the five time points. Ten dominant classes, including Deltaproteobacteria (12.08%), Bacteroidia (6.06%), Anaerolineae (4.52%), *Candidatus* Cloacamonas (3.21%), Methanomicrobia (2.80%), Clostridia (2.26%), Ignavibacteria (1.80%), Synergistia (1.11%), Thermotogae (0.98%), and Gammaproteobacteria (0.8%) were revealed in all samples. And most of these dominant classes had no considerable temporal relative abundance variation, except for Gammaproteobacteria which with relative abundance of 1.52 and 1.67% in T2 and T3 but with very low relative abundance of 0.32, 0.32, and 0.14% in T1, T4, and T5 (**Figure 7A**).

Out of the 92 orders revealed by this study, 13 dominant orders were Bacteroidales (6.06%), Anaerolineales (4.52%), Desulfovibrionales (3.43%), *Candidatus* Cloacamonas (3.21%), Desulfuromonadales (3.14%), Methanosarcinales (2.45%), Clostridiales (2.18%), Ignavibacteriales (1.80%), Syntrophobacterales (1.67%), Synergistales (1.11%), Kosmotogales (0.98%), Flavobacteriales (0.50%), and Aeromonadales (0.45%). Among them, the most abundant orders maintained high stabilization with time changing, only some minor abundant orders presented some temporal variation. For instance, the relative abundance of Flavobacteriales was higher in T3 (0.99%) and T4 (1.02%) than other three time points (0.03, 0.35, and 0.12% in T1, T4, and T5, respectively). And another order Aeromonadales held higher relative abundance in T2 (1.04%) and T3 (1.12%), but appeared as rare species in T1 (0.03%), T4 (0.06%), and T5 (lower than 0.01%) (**Figure 7B**).

**FIGURE 8:**
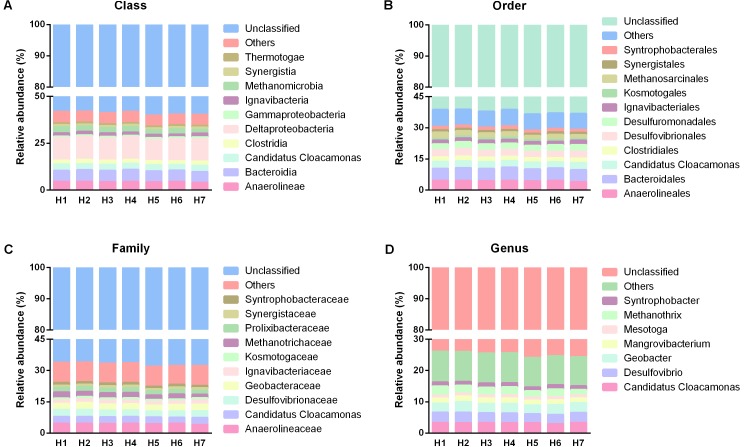
Taxonomic distribution of different sampling heights from class to genus. **(A–D)** Were the taxonomic information of predominant populations in class, order, family and genus level, respectively.

In term of family level, 10 dominant families were observed in the total 160 families (**Figure 7C**). The most dominant families belonged to Anaerolineaceae (4.52%), Desulfovibrionaceae (3.29%), *Candidatus* Cloacamonas (3.21%), Geobacteraceae (3.14%), followed by Methanotrichaceae (2.45%), Prolixibacteraceae (2.26%), Ignavibacteriaceae (1.8%), Syntrophobacteraceae (1.31%), Synergistaceae (1.11%), and Kosmotogaceae (0.98%). The overall distribution of these families showed high temporal similarity among the five time points. At genus level, only eight dominant genera, including *Desulfovibrio* (3.26%), *Candidatus* Cloacamonas (3.21%), *Geobacter* (3.14%), *Methanosaeta* (2.45%), *Mangrovibacterium* (1.74%), *Syntrophobacter* (1.30%), *Mesotoga* (0.98%), and *Pelolinea* (0.90%) were observed in all the samples, and they demonstrated similar temporal distribution with that in family level (**Figure 7D**). For the spatial taxonomic distribution, all the taxa from phylum to genus also presented extraordinary spatial uniformity in different sampling heights (**Figures 4B**, **8A–D**) and the major phylogenetic group composition kept similar in temporally.

All above evidence proved that although some tiny variation could be observed in a few phylogenetic groups, they were not the predominant members. The high abundant major taxa sustained the temporal stability of microbial community structure. Spatial homogeneity of microbial community structure was observed in the AnaEG reactor. The characteristics of microbial distribution were highly consistent with the reactor’s temporal treatment performance.

## Discussion

The treatment performance of the AnaEG reactor indicated a high efficiency both in temporal and spatial scale. For the temporal treatment performance evaluation, many studies showed that the quality of wastewater could affect the treatment efficiency of anaerobic bioreactor. For example, in a long-term study, when the mesophilic biofilm-based EGSB was used to starch-containing wastewater treatment and simultaneous hydrogen recovery, only low treatment performance was completed (average COD removal rate was 31.1%) when it operated at the OLR of 0.125–1 g starch L^-1^ d^-1^, pH of 3.68–4.95, and HRT of 4–24 h. Methanogens is known as a group of microorganisms who are sensitive to pH changes, low pH may be the important reason of their low COD removal rate (Guo et al., 2008). In another report, short pH shock (pH decreased from 9.0 to 7.0 for 24 h) could lead a deterioration of EGSB (He et al., 2016). But in our study, the AnaEG reactor could always implement desirable treatment efficiency with the average COD removal rate higher than 95%, although with variable influent, such as shock pH (5.7–8.5) and OLR (1–3.3 kg COD m^-3^ d^-1^). The results indicated the microbial community in our bioreactor was a resilient ecosystem and could maintain a strong functional stabilization when it treated the varied influent.

Moreover, it was worth considering why the pollutants was mainly degraded at the bottom of this AnaEG reactor. In previous research (Lu et al., 2015), the main reaction zone for starch hydrolysis, located between the bottom and middle areas of the UASB and the organic compounds gradually attenuated along with the upflow. The different main reaction zones in these researches may due to the structural design difference of the reactor. Because AnaEG is a plug flow pattern reactor which ensures the sufficient expansion of sludge bed. This particular design improved the contact between substrate and microorganism, and reinforced the transfer effect. In addition, VFAs analysis revealed that starch was directly degraded to acetate, and very few intermediates such as propionate and butyrate produced, which is consistent with previous research (Schmidt and Ahring, 1996). Consequently, organic matter could be degraded efficiently at the bottom area, and high treatment performance could be completed even though if with the transient influent. Furthermore, in the study previously described, smaller granules had higher nitrogen removal rate than the larger ones (Wang et al., 2014), the former offered more sufficient contact area between substrate and microorganism to complete preferable treatment efficiency. The granule size in our study, obviously smaller (0.9 mm) than that of ever previous described SPW anaerobic treatment reactors (2–5 mm) (Nizami and Murphy, 2011; Lu et al., 2015). This characteristic of the AGS further strengthen the AnaEG treatment efficiency. More important, the AnaEG offered a possibility to the industrial application for a high efficient low organic loading wastewater processing, although the anaerobic reactor was traditionally recognized as especially applicable for high organic loading wastewater treatment.

In term of microbial community of AnaEG, the species diversity was much higher than other anaerobic bioreactor. For example, in a thermophilic anaerobic digestion of pig slurry, the Shannon index only ranged from 3.34 to 4.76, and the maximum COD removal rate was less than 60% (Cerrillo et al., 2017). In a continuous stirred tank reactor which fed with distiller’s grains and supplemented with trace elements, the Shannon index of the reactor was only 4.47–4.56 (Wintsche et al., 2016). Although other study reported a relatively higher species diversity in starch wastewater, such as in the description of Antwi et al. (2017), they found the Shannon index in different section of UASB ranged from 4.61 to 5.26 when it used to potato starch wastewater treatment. But all of them were much lower than the species diversity of AnaEG reactor in this study, the Shannon index of this reactor were ranged from 6.28 to 6.63, and its COD removal rate kept above 90%. Therefore, the higher species diversity might be one important reason of the high efficiency of AnaEG reactor.

The temporal stable and spatial uniform microbial community structures in AnaEG reactor showed remarkable agreement with the reactor’s performance. But an obvious spatial distribution was observed in a similar experiment of lab-scale EGSB (Ambuchi et al., 2016), in which Chloroflexi, Euryarchaeota, and Firmicutes were the dominant phyla in the reactor’s bottom, middle, and upper segments, respectively. However, no operation performance data were available to illustrate the reasons for the microbial community spatial distribution. Moreover, substantial stratification of the microbial community was also observed when the UASB in potato starch wastewater processing had a shorter HRT (36 h; Antwi et al., 2017). It appeared that shortening the HRT may lead to microbial spatial hierarchical distribution, and this was also shown in a previous study (Lu et al., 2015). Nevertheless, the organic matter concentration was low and kept stable from the first port to the upper port of the AnaEG in this study, and also the bacterial composition was unchanged along the vertical direction. The possible reason of this phenomenon may be caused by efficiently gas stirring which produced in anaerobic digestion under the special plug flow pattern of AnaEG. Thus, the interior of the reactor could be a uniform system. Therefore, no spatial hierarchical distribution could be observed.

Because the SPW is a common biodegradable wastewater, it is reasonable that we regarded the predominant populations as the authentic functional performers and it should be responsible for the temporal treatment stabilization of the AnaEG. In our study, we comprehensively investigated the temporal and spatial distribution of microbial community structure on OTU level. All the results implied that high abundant microorganisms consist of the main part of the overall microbial community. This part microorganisms should be functional crucial members. And thanks to their temporal stability, the AnaEG reactor could implement temporal stable and high efficient treatment performance.

Furthermore, some of the predominant phyla detected in this study, such as Bacteroidetes, Chloroflexi, Euryarchaeota, Firmicutes, and Proteobacteria were also considered as predominant microorganisms in sugar industrial wastewater treatment (Ambuchi et al., 2016). And because of the high biodegradable ability of starch, many hydrolytic and acidification reactions need to attend the anaerobic biodegradation. As expected, all the dominant phyla detected in this study were generally recognized as common anaerobic fermentation phyla (Zhou et al., 2016). Chloroflexi (Lynd et al., 2002) and Firmicutes (Bertin et al., 2012) were often involved as critical anaerobic hydrolytic and acidifying phyla. The bacteria of the dominant genus of *Mesotoga* in this study had been considered to be hydrolytic bacteria (Imachi et al., 2014), and *Syntrophobacter* seemed to possess a higher affinity to degrade propionate (Gomes et al., 2016). These literatures further supports our point that the dominant community was composed of hydrolytic and acidifying microorganisms. Notably, the microorganisms of *Methanosaeta* which comprised most of the methanogens investigated in this work, was recognized as aceticlastic methanogens (Imachi et al., 2014; Rotaru et al., 2014). *Methanosaeta* was also reported to be the predominant methanogens in a study that used lab-scale UASB to treat potato SPW (Antwi et al., 2017). Considered that acetic acid was one of the major component in the SPW, and the predominant role of *Methanosaeta* in the reactor, we may concluded that the aceticlastic pathway predominated the methanogenesis in AnaEG reactor. Besides, many multifunctional degradable microorganisms that are common in wastewater were detected in the AnaEG reactor, such as *Thauera*, it was not only widely regarded as a kind of functional bacteria with denitrification, but also could degrade aromatic compounds (Wu et al., 2015). In other words, the reactor may have the potential to other complex compounds degradation.

## Conclusion

In this study, the temporal and spatial evaluation of the operation performance and microbial community structure of an AnaEG revealed that, the reactor operated with high efficiency and high temporal stability. The predominant populations, including Bacteroidetes, Chloroflexi, Firmicutes, and Proteobacteria, and *Methanosaeta*, carried out the metabolism of anaerobic hydrolysis, acidification, and methanogenesis. And this part of functional microorganisms determined the overall microbial community characteristics. No temporal variety and spatial hierarchy of microbial community structure had been detected in the AnaEG reactor. And the microbial community dynamics displayed remarkable coherence with the reactor’s performance.

## Author Contributions

XQ, XW, and LL performed the experiments. XZ, ZZ, and CL contributed to the conception and design of the study. XQ wrote the first draft of the manuscript, and all the authors contributed to manuscript revision and approved the final manuscript.

## Conflict of Interest Statement

The authors declare that the research was conducted in the absence of any commercial or financial relationships that could be construed as a potential conflict of interest.

## Footnotes

1 http://rdp.cme.msu.edu/classifier/classifier.jsp
