# Supplementary material for: The Advanced Anaerobic Expanded Granular Sludge Bed (AnaEG) Possessed Temporally and Spatially Stable Treatment Performance and Microbial Community in Treating Starch Processing Wastewater
Source: Front Microbiol. 2018 Mar 28;9:589. doi: 10.3389/fmicb.2018.00589 (PMC5882818; doi:10.3389/fmicb.2018.00589)
Supplement: Supplementary file 1 [file Presentation_1.PDF]

Supplementary Information

Figure Captions

Figure S1. Complete picture of advanced anaerobic expanded granular sludge bed (AanEG). (A) The full view of AnaEG reactor. (B) The upper view of AnaEG. (C) Seven sampling ports (H1-H7) of AnaEG.

Figure S2. SEM observation of AGS morphological characteristics. H1 to H7 represented granules of seven sampling ports.

Figure S3. Alpha diversity of AGS. (A) Shannon index curve of all samples. (B) Rarefaction curve of all samples.

Figure S4. Temporal and spatial characteristics of Shannon diversity. Error bars were SD, ANOVA analysis of Kruskal-Wallis test was used to analyze variation between different groups, $^{**}P < 0.01$.

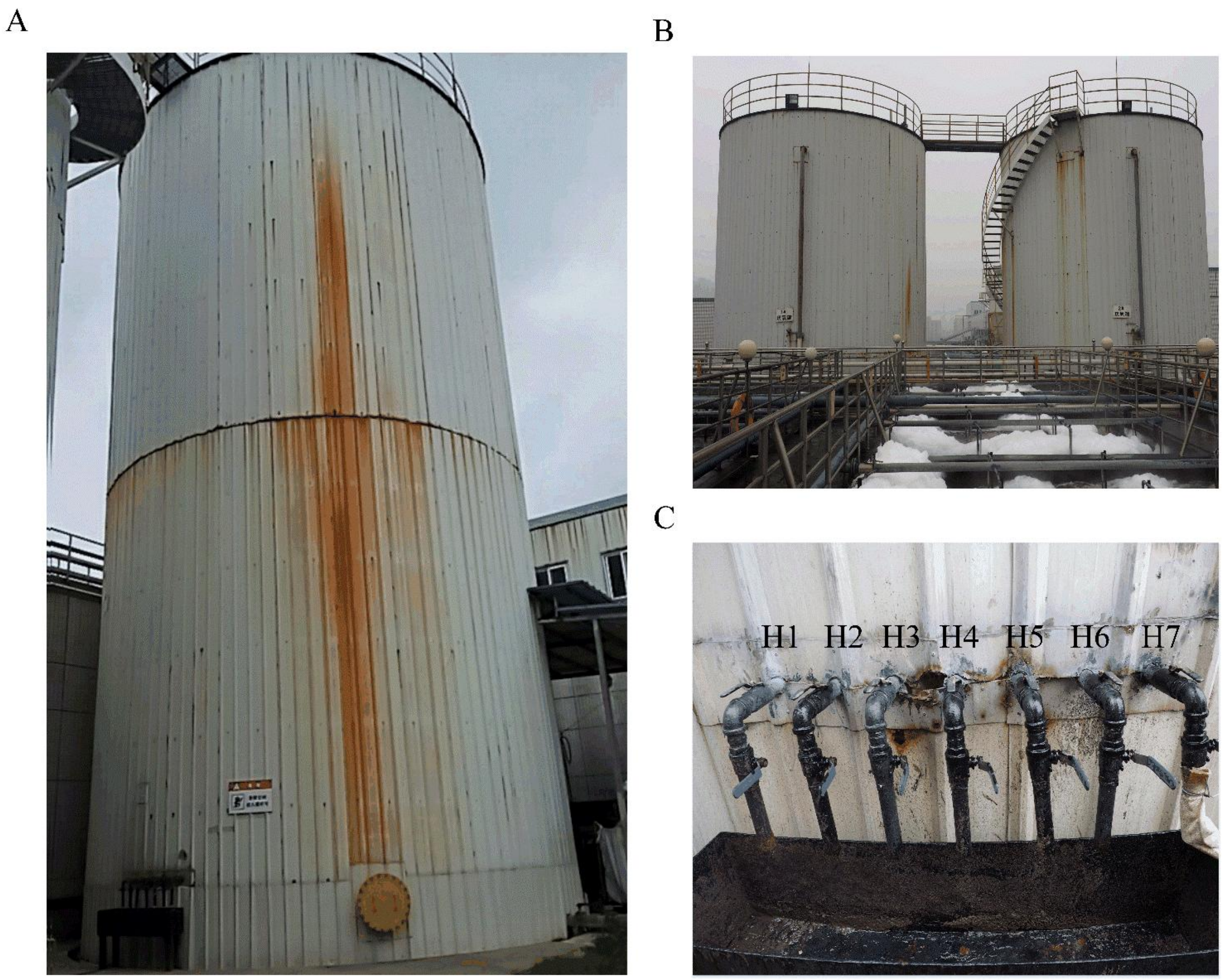

Figure S1. Complete picture of advanced anaerobic expanded granular sludge bed (AanEG). (A) The full view of AnaEG reactor. (B) The upper view of AnaEG. (C) Seven sampling ports (H1-H7) of AnaEG.

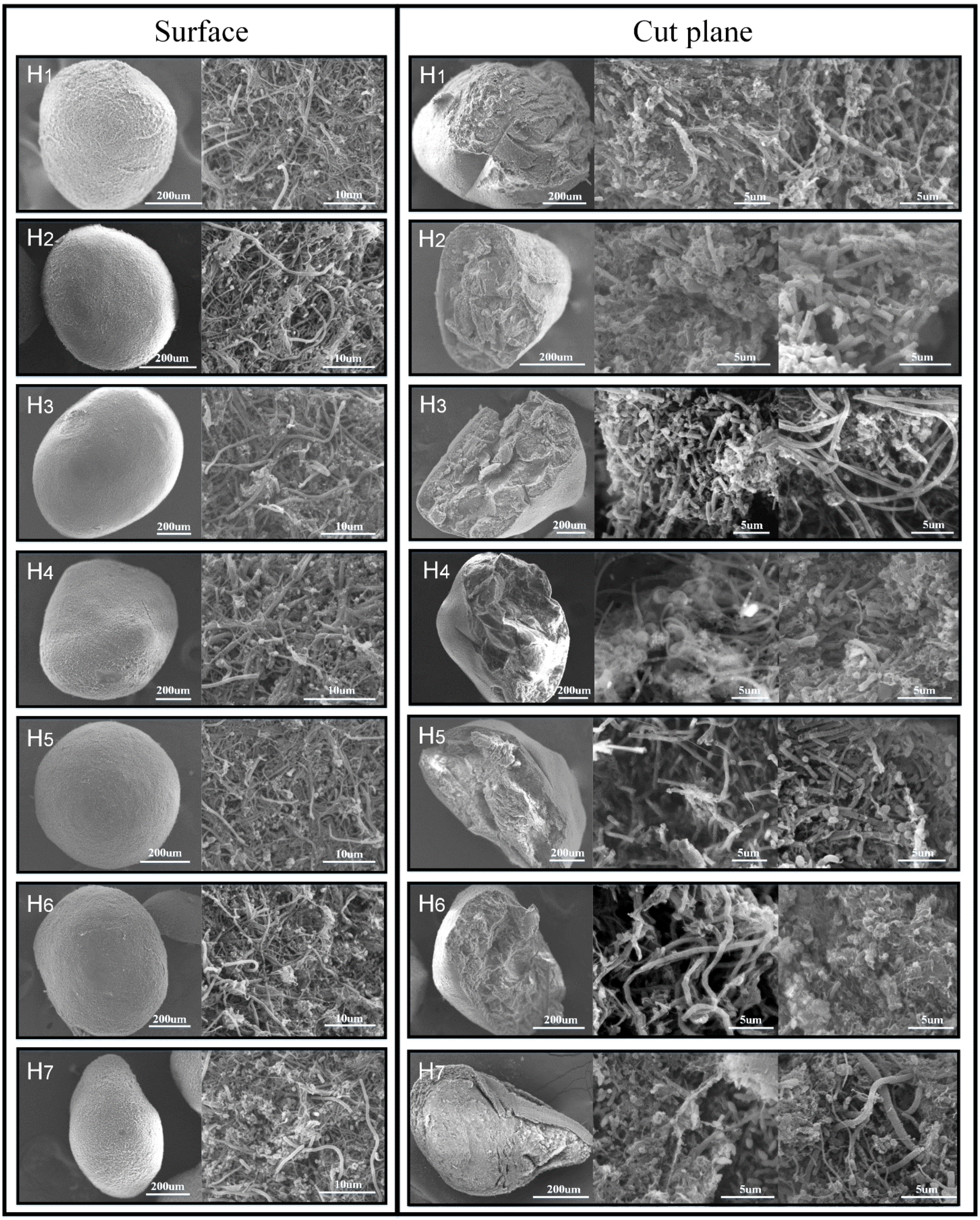

Figure S2. SEM observation of AGS morphological characteristics. H1 to H7 represented granules of seven sampling ports.

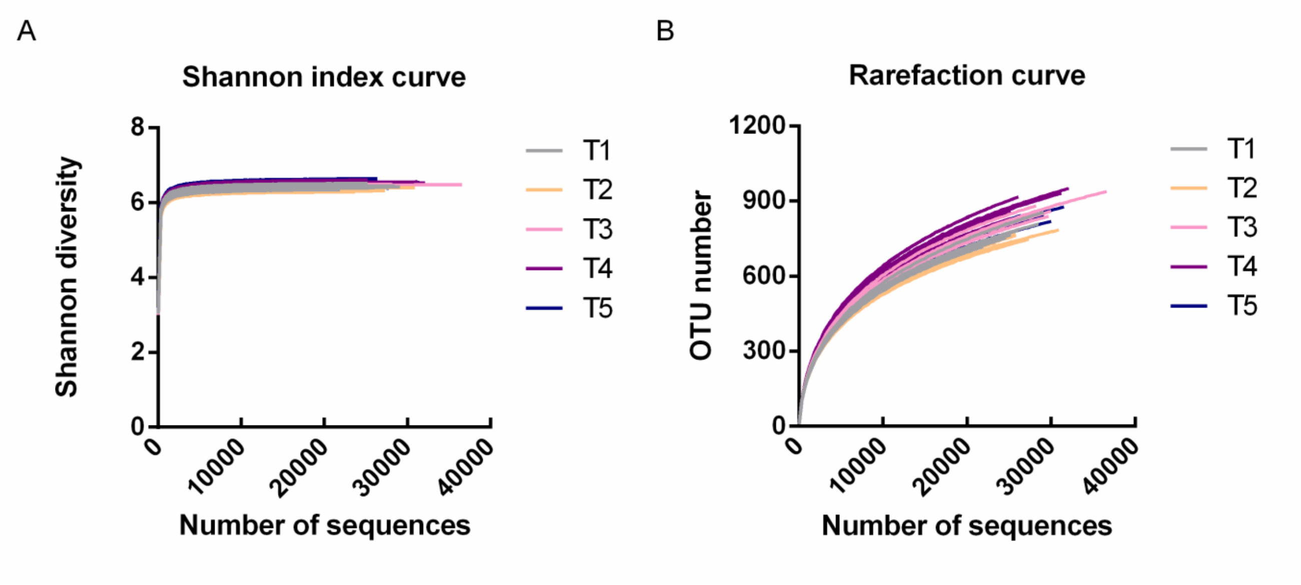

Figure S3. Alpha diversity of AGS. (A) Shannon index curve of all samples. (B) Rarefaction curve of all samples.

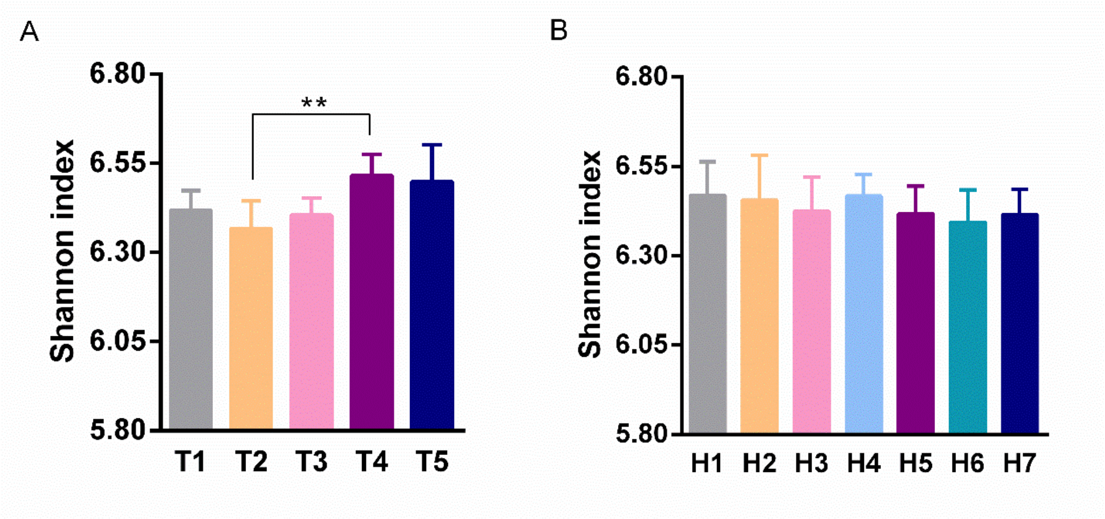

Figure S4. Temporal and spatial characteristics of Shannon diversity. Error bars were SD, ANOVA analysis of Kruskal-Wallis test was used to analyze variation between different groups, $^{**}P < 0.01$.
